# Recent advances in phosphorescent platinum complexes for organic light-emitting diodes

**DOI:** 10.3762/bjoc.14.124

**Published:** 2018-06-18

**Authors:** Cristina Cebrián, Matteo Mauro

**Affiliations:** 1 Université de Lorraine, CNRS, L2CM, F-57000 Metz, France; 2 Université de Strasbourg, CNRS – Institut de Physique et Chimie des Matériaux de Strasbourg (IPCMS), UMR 7504, 23 rue du Loess, F-67000 Strasbourg, France

**Keywords:** cyclometalating ligands, electroluminescence, OLED, phosphorescence, platinum complexes

## Abstract

Phosphorescent organometallic compounds based on heavy transition metal complexes (TMCs) are an appealing research topic of enormous current interest. Amongst all different fields in which they found valuable application, development of emitting materials based on TMCs have become crucial for electroluminescent devices such as phosphorescent organic light-emitting diodes (PhOLEDs) and light-emitting electrochemical cells (LEECs). This interest is driven by the fact that luminescent TMCs with long-lived excited state lifetimes are able to efficiently harvest both singlet and triplet electro-generated excitons, thus opening the possibility to achieve theoretically 100% internal quantum efficiency in such devices. In the recent past, various classes of compounds have been reported, possessing a beautiful structural variety that allowed to nicely obtain efficient photo- and electroluminescence with high colour purity in the red, green and blue (RGB) portions of the visible spectrum. In addition, achievement of efficient emission beyond such range towards ultraviolet (UV) and near infrared (NIR) regions was also challenged. By employing TMCs as triplet emitters in OLEDs, remarkably high device performances were demonstrated, with square planar platinum(II) complexes bearing π-conjugated chromophoric ligands playing a key role in such respect. In this contribution, the most recent and promising trends in the field of phosphorescent platinum complexes will be reviewed and discussed. In particular, the importance of proper molecular design that underpins the successful achievement of improved photophysical features and enhanced device performances will be highlighted. Special emphasis will be devoted to those recent systems that have been employed as triplet emitters in efficient PhOLEDs.

## Introduction

Photoactive TMCs have attracted enormous attention in the last two decades because of their peculiar photophysical and rich redox properties, which make them appealing from both fundamental research and technological applications points of view. Nowadays, several research groups have devoted much effort in exploring a large variety of classes of luminescent TMCs with closed-shell d^6^, d^8^ and d^10^ electronic configurations [1–5]. The concomitant presence of a heavy metal ion and coordinated π-conjugated chromophoric ligands enriches the photophysical features displayed by TMCs when compared to classical organic luminophors. Indeed, apart from ligand centred (LC) and intraligand charge transfer (ILCT) states, admixing of the metal and ligand orbitals close to the frontier region results in excited states featuring a certain degree of metal contribution. In particular, metal-to-ligand charge transfer (MLCT), ligand-to-metal charge transfer (LMCT), ligand-to-ligand charge transfer (LLCT) and metal centred (MC) states actively contribute to the richer photophysical and photochemical features of TMCs and to their resulting properties, also in terms of electrochemistry. Additionally, the presence of a heavy metal atom induces spin-orbit coupling (SOC) effects to such an extent that intersystem crossing (ISC) processes become thus competitive over other radiationless deactivation pathways owing to relaxation of spin rules. In this way, long-lived and low energy lying excited states with triplet (T_n_ states) character are accessible and can be efficiently populated. The subsequent deactivation from the lowest lying T_1_ state into the electronic ground state (S_0_) through radiative channels, T_1_ → S_0_, occurs with decay kinetics between hundreds of nanoseconds to several microseconds, constituting a formally spin-forbidden transition (phosphorescence). Structural modification of the TMCs and proper tailoring of coordinated ligands can independently act on the nature, energy and topology of frontier orbitals. In fact, a fine modulation is achieved through a precise energetic positioning and mixing of different excited states, as well as tuning of the energetic band gap between S_0_ and the lower-lying singlet and triplet manifold excited states. This approach did successfully yield phosphorescent TMCs with an emission wavelength tuneable over the entire visible spectrum and beyond; together with compounds with photoluminescence quantum yield (PLQY) approaching unity. These peculiar features have greatly fuelled the still growing interest in luminescent TMCs for its potential employment in applications and real-market technology including photocatalysis [6], bio-imaging [7–8], and solar-energy conversion [9], just to cite a few.

Thompson and Forrest reported in 1998 on the first example of a phosphorescent emitter, namely 2,3,7,8,12,13,17,18-octaethyl-21*H*,23*H*-porphyrin platinum(II) (Pt(OEP)), used as dopant for the fabrication of an efficient (external quantum efficiency, EQE, ca. 4%) OLED device [10]. Since that pioneering work, an impressive amount of research effort has been devoted in the last two decades to seeking for TMCs that display better device performances. In this respect, iridium(III) and platinum(II) derivatives undoubtedly play leading roles as electro-active materials in light-emitting devices. Their outstanding photophysical and electrochemical features enabled fabrication of PhOLEDs and LEECs [11] with enhanced device performances in terms of efficiency, operating lifetime and colour purity. In electrophosphorescent devices, the triplet nature of excited states localized on the active TMCs allows harvesting of both singlet and triplet electro-generated excitons through either direct trapping or energy transfer processes. As a consequence, the theoretical internal quantum efficiency rises from 25%, which corresponds to purely fluorescent-based devices from a first approximation spin statistics, up to 100%. Nonetheless, EQEs are typically upper limited to values of ca. 20–25% owing to differences in the refractive index of organic materials commonly employed and suboptimal light outcoupling. In spite of that, highly performing vacuum-processed devices with record EQEs up to 54% have been reported to date for PhOLEDs based on Ir(III) with optimized light outcoupling [12]. On the other hand, an impressive EQE value as high as 38.8% [13] and 55% [14] have been recently achieved in platinum(II)-based OLEDs without and with outcoupling elements, respectively, via engineering of transition dipole moment orientation in the device active matrix.

Owing to the enormous interest they are currently attracting, the scope of the present review article is to highlight the current trends and achievements in the field of phosphorescent platinum complexes for PhOLEDs with a special emphasis on the most recent advances. It should be noted that this contribution is not indented to be comprehensive and readers are invited to refer elsewhere for previous examples of platinum emitters [15–18]. In particular, we will focus our attention on recently reported Pt(II) complexes by breaking down the different classes into those containing monodentate, bidentate, tridentate and tetradentate chromophoric ligands, in order to put in context and compare their photophysical and electroluminescent properties. Finally, some very recent and interesting examples of Pt(IV) compounds as triplet emitters in OLEDs, a class of compound that has been much less explored, will also be reviewed. PhOLED performances of devices comprising the examples reviewed herein are summarised in Table 1.

## Review

### Platinum(II) complexes

#### Platinum complexes bearing mono-dentate ligands

**Figure 1:**
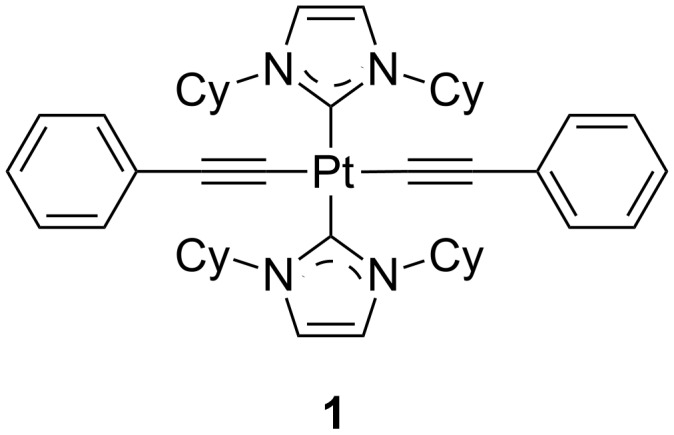
Molecular structure of neutral platinum(II) complex **1** bearing four monodentate ligands; cy = cyclohexyl [19].

Platinum(II) complexes bearing monodentate ligands are likely to have very poor luminescent properties. In these complexes, the molecular flexibility as a consequence of the low denticity favors efficient thermal deactivation via MC excited states and other nonradiative relaxation pathways. Schanze and co-workers have demonstrated, however, that it is possible to obtain satisfactory photo- and electroluminescence from *trans*-platinum(II) complex **1** bearing only monodentate ligands (Figure 1) [19]. In this derivative, the MC states were efficiently destabilized by selecting strong σ-donating NHC and –C≡C–R ligands. The presence of the two bulky cyclohexyl substituents on the imidazolylidene moiety contributed to rigidify the structure, as well as avoid detrimental intermolecular interactions. Though being weakly emissive in THF solution, the compound exhibited a narrow deep blue photoluminescence (CIE = 0.14, 0.12) with a PLQY of 0.30 in PMMA films. Multilayer vacuum-processed OLEDs were fabricated to test the electroluminescence performance of this complex. A remarkable value of 8% of EQE was attained, but a severe roll-off efficiency was observed with an EQE value dropping to 2% at a practical brightness of 500 cd m^−2^. Nevertheless, this work opens the door for a novel design of highly efficient deep-blue phosphors.

**Figure 2:**
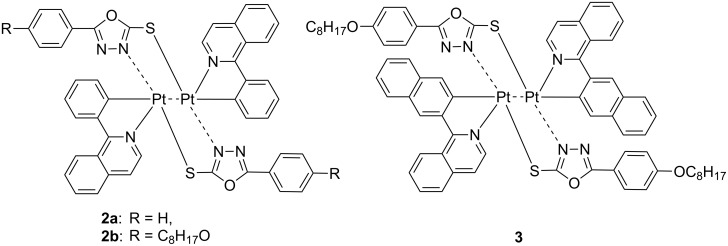
Chemical structure of the dinuclear Pt complexes **2a–b** and **3** [20].

More complex structures based on dinuclear platinum(II) complexes have also been recently described [20]. Upon using two 1,3,4-oxadiazole-2-thiol as bridging ligands coordinating two Pt(II) centers in a monodentate fashion, Zhu and co-workers have reported on dimeric structures, namely **2** and **3**, exhibiting an interaction between the two metallic centres (Pt···Pt distance of ca. 3 Å) (Figure 2). The appearance of a triplet metal–metal-to-ligand charge transfer (^3^MMLCT) transition led to NIR emission with PLQY of ca. 0.31. These bimetallic compounds were tested as dopants in solution-processed PLED, achieving EQE values up to 5.2% at 100 mA cm^−2^, even though with relatively high turn-on voltages of 10.4–14.6 V. However, molecular aggregation was observed at dopant concentrations above 12 wt %.

Although (hetero-)metallic clusters are beyond the scope of this review, it is worth to mention some recent reports from Chen and co-workers on trimetallic systems based on PtAu_2_ [21–22] and PtAg_2_ [23–24] core. Motivated by very high PLQYs in doped films, OLED devices were fabricated showing remarkable efficiency attaining EQE of 21.5% at a luminance of 1029 cd m^−2^ with small roll-off [21]. These performances are the best reported so far for such a practical luminance.

#### Systems based on bidentate ligands

In the past, the most common synthetic strategy to obtain luminescent platinum(II) complexes has been the use of π-conjugated chelating ligands with a bidentate motif bearing π-accepting (hetero)aromatic units. Compared to monodentate ligands, the more rigid structure of the bidentate motif is expected to reduce excited-state molecular distortion and access to quenching channels to some extent. On the other hand, the appearance of new low-lying excited states associated to the π molecular orbitals typically results into efficient emission due to their larger radiative decay rates [25].

**Figure 3:**
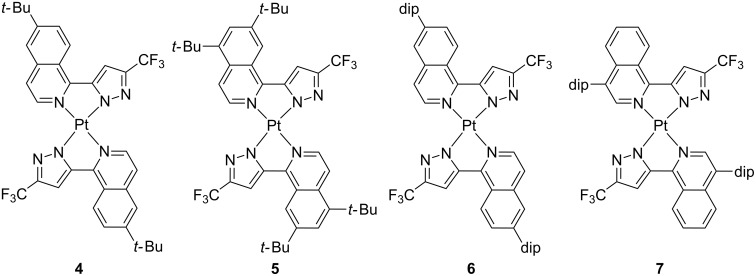
Molecular structure of platinum(II) complexes bearing isoquinolinylpyrazolates; dip = 2,6-diisopropylphenyl [26].

Though limited in the 1980s by their sensitive synthesis via lithiated species, archetypical luminescent platinum(II) complexes were based on 2-phenylpyridine (ppy) and its derivatives. The combination of the strong σ-donor effect of the phenylate and the π-accepting character of the pyridine ring results in a high ligand-field for the coordinated metal, thus raising the energy of quenching d–d states while lowering emissive MLCT and LC excited states. Alternatively, the use of *N*-deprotonable azole units has also been largely explored due to the fact that it can exert similar effects to ppy-like ligands [16]. Nevertheless, easier deprotonation of the N–H site in comparison with ppy chelates notably widens applicability and increases the chemical structure diversity of the final luminophors, e.g., for complexating metal ions less prone to undergo cyclometallation reactions. Extensive work based on azolate-type of ligands has been developed by the group of Chi [16] who has recently described a series of neutral platinum(II) complexes bearing isoquinolylpyrazolates, complexes **4**–**7** in Figure 3 [26]. Control on the intermolecular interactions was exerted through the substitution pattern, yielding solids that exhibited mechano- and solvatochromic properties. Indeed, bathochromic shifts in the emission energy were observed upon either grinding or incrementing solvent polarity. This emission was attributed to a radiative transition with triplet metal–metal-to-ligand charge transfer character (^3^MMLCT), which ultimately strongly depends on the platinum···platinum intermolecular distance. These compounds were also suitable OLED dopants, achieving high EQE of 8.5–11.5%. Nevertheless, the electroluminescence was slightly broader than the corresponding photoluminescence due to incomplete suppression of the intermolecular interactions.

**Figure 4:**
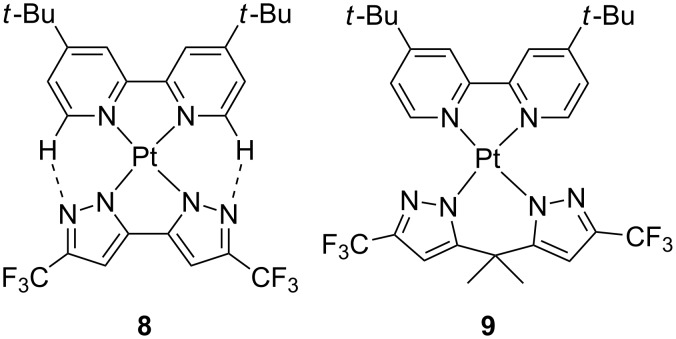
Selected neutral platinum(II) complexes featuring dianionic biazolate and neutral bipyridines [27].

Taking advantage of the easy generation of anionic ligands from azoles, the same group described the preparation of neutral platinum(II) complexes resulting from the combination of dianionic with neutral chelates (Figure 4) [27]. Compounds **8** and **9** were weakly emissive in solution. Nevertheless, the solid-state emission of these particular heteroleptic complexes was switched on notably. Apart from reduced geometry distortions within a rigid environment, the presence in some cases of interligand H-bonding interactions further contributed to efficiently suppress nonradiative decay channels. More importantly, these supplementary interactions reinforced the ligand–metal bond, which explains well the remarkable phosphorescence efficiency obtained in solid-state thin films being PLQY of 0.52 and 0.83 for **8** and **9**, respectively. Such findings prompted the authors to fabricate non-doped OLEDs with an architecture as follows: ITO/MoO_3_ (2 nm)/1,4-bis(1-naphthylphenylamino)biphenyl (NPB) (25 nm)/1,3-bis(9-carbazolyl)benzene (mCP) (8 nm)/complex **8** or **9** (40 nm)/tris[3-(3-pyridyl)mesityl]borane (3TPyMB) (50 nm)/LiF (1 nm)/Al. The OLED based on **8** displayed orange-red electroluminescence (EL) with EQE of 19.0%, current efficiency (CE) of 21.0 cd A^−1^, power efficiency (PE) of 15.5 lm W^−1^ and brightness as high as 43000 cd m^−2^. On the other hand, yellow emitting OLED were obtained for **9** with EQE of 7.1%, CE of 21.0 cd A^−1^, PE of 11.3 lm W^−1^ and brightness of 5100 cd m^−2^. The better performances of **8** over **9** were ascribed to a shorter exciton lifetime that contributes to reduce detrimental nonradiative processes such as triplet–triplet annihilation (TTA) and triplet–polaron annihilation (TPA).

**Figure 5:**
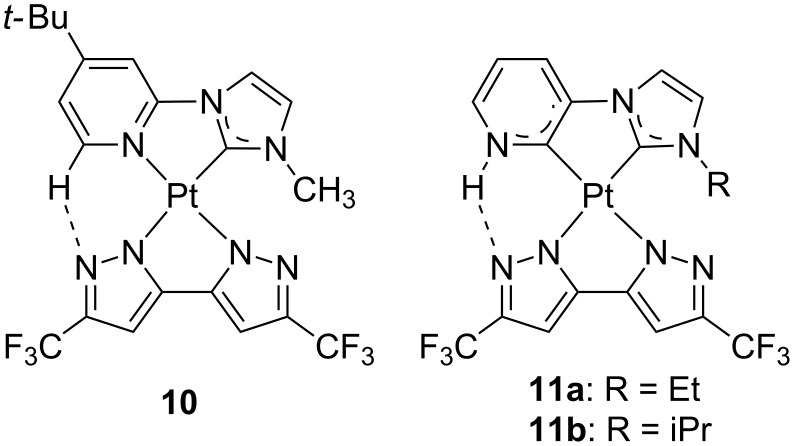
Selected neutral platinum(II) complexes from bipyrazolate and carbene-based chelates [34–35].

On the other hand, strong σ-donor NHC carbenes ligands could be regarded as the neutral variant of phenylate-like counterparts [28–30]. Apart from the strong σ-donor ability, the great interest for these ligands relies on the robustness that they confer to the resulting complexes, upon coordination onto both early [31] and late transition metals [32–33]. In this regard, the group of Chi employed carbene-based chelates as neutral imine substitutes in an attempt to further improved the stability and the performances of their N···H–C stabilized phosphors (Figure 5) [34–35]. Either when one, compound **10** [34], or two, compound **11** [35], carbene moieties were used, the resulting platinum compounds were basically nonemissive in solution. On the contrary, they became strong emitters in the solid state owing to the switching of the nature of the excited state that becomes ^3^MMLCT in nature. Their EL properties were evaluated by fabrication of non-doped OLEDs. Compound **10** was embedded into an OLED device with the following configuration ITO/MoO_3_ (1 nm)/TAPC (65 nm)/mCP (8 nm)/**10** (pure/nondoped, 30 nm)/3TPYMB (50 nm)/LiF (1 nm)/Al (120 nm), where TAPC is 1,1-bis[(di-4-tolylamino)phenyl]cyclohexane, and serve either as the hole- or electron-transport layers. A highly efficient yellow-emitting device was obtained with EQE = 25.9% and CE = 90 cd A^−1^ at 100 cd m^−2^ (EQE = 24.4%, CE = 85 cd A^−1^ at 1000 cd m^−2^); one of the best performances ever reported for a non-doped OLED. On the other hand, device architecture for compound **11** was as follows: ITO/TAPC with 20 wt % MoO_3_ (20 nm)/TAPC (40 nm)/2,6-bis(3-(9*H*-carbazol-9-yl)phenyl)pyridine (26DCzppy) with 8 wt % of **11** (20 nm)/1,3,5-tris[(3-pyridyl)phen-3-yl]benzene (TmPyPB) (50 nm)/LiF (0.8 nm)/Al (150 nm). The associated OLED performances for **11** were lower respect to those of the former compound, yielding a green-yellow emission with EQE = 12.5%, CE = 44.0 cd A^−1^ and PE = 28.0 lm W^−1^ for **11a**, and EQE = 11.2%, CE = 40.6 cd A^−1^ and PE = 25.8 lm W^−1^ for **11b**. In consequence, the use of only one carbene moiety seemed to afford very appealing photophysical features both for display and lighting applications.

**Figure 6:**
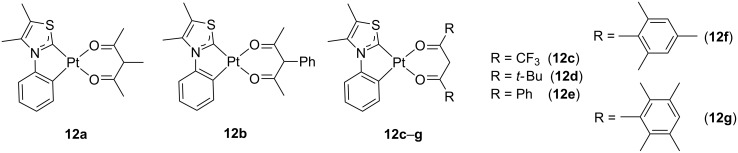
Cyclometalated thiazol-2-ylidene platinum(II) complexes with different acetylacetonate ligands [37].

The beneficial effect of carbene moieties on the photophysical features of the dopant was also shown by Strassner and co-workers [36–38]. Compared with previously reported imidazolylidene and triazolylidene acetylacetonate (acac) platinum(II) complexes, complexes **12** bearing 1,3-thiazol-2-ylidene carbenes outperformed the former when evaluating the photophysical properties (Figure 6) [37]. The intermolecular interaction was finely tuned as a function of the steric hindrance of the acac-type ancillary ligand, which had a profound impact on the emission quantum yield. Characterization of the electroluminescence performances of these complexes in mixed-matrix OLED led to EQE values as high as 12.3%, CE of 37.8 cd A^−1^ and PE of 24.0 lm W^−1^ at 300 cd m^−2^ for complex **12f**.

**Figure 7:**
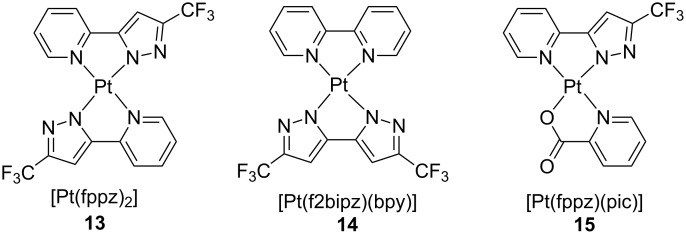
Neutral platinum(II) complexes **13**–**15** bearing azolate ligands [13].

In spite of typical TTA processes at high concentrations for phosphorescent dopants, azolate-containing platinum(II) complexes have recently shown great potentiality for the fabrication of non-doped OLEDs. In fact, Wang and collaborators reported a red-emitting device based on Pt(fppz)_2_ [39], where fppz is 3-(trifluoromethyl)-5-(2-pyridyl)-1*H*-pyrazolate, that attained remarkable EQE of 31% [40] (see Figure 7 for the chemical structure of the complex). With the aim of correlating molecular structure, photophysical properties and OLED performances, Chi, Kim and co-workers analyzed the X-ray structures of Pt(fppz)_2_ (**13**) and other related platinum(II) complexes **14** and **15** in both single crystal and thin film samples (Figure 7) [13]. They observed different degrees of crystallinity as a function of the substrates, though the crystal pattern of the investigated compounds was not affected. More interestingly, upon analysis of angle-dependent emission intensities at various wavelengths along with the birefringence of the films, the authors concluded that the arrangement of the complexes within the films was crucial for the PLQY attained. In the remarkable case of the crystalline film of complex Pt(fppz)_2_, the molecular plane of the square-planar compound was mostly perpendicular respect to the substrate and hence, the ^3^MMLCT photoluminescence dipole lies almost parallel to it. The architecture of the fabricated OLEDs using phosphor **13** as emitting layer was ITO (100 nm)/TAPC (80 nm)/4,4’,4’’-tri(9-carbazoyl)triphenylamine (TCTA) (10 nm)/Pt(fppz)_2_ neat (30 nm)/1,3-bis(3,5-di(pyridin-3-yl)phenyl)benzene (BMPYPB) (15 nm)/BMPYPB:1 wt % Rb_2_CO_3_ (40 nm)/Al (100 nm). These devices exhibited an outstanding EQE value as high as 38.8%, which approaches the maximum EQE estimated value of ca. 45%. This latter could be achieved in the case of a phosphor with 100% of PLQY with a fully parallel emitting dipole.

**Figure 8:**
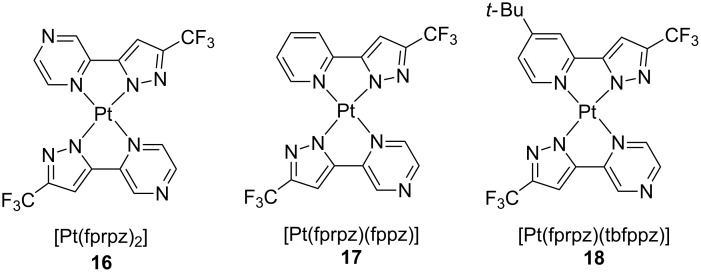
Chemical structure of neutral platinum(II) complexes **16**–**18** bearing azine-pyrazolato bidentate ligands [14].

The beneficial effect of the emitting dipole orientation on the light outcoupling efficiency was further illustrated in a following work by the group of Chi [14]. Exploiting the strong tendency to form ordered structures, a new series of platinum(II) bearing fluorinated 2-pyrazinylpyrazoles was developed, namely complexes **16**–**18** in Figure 8. Upon aggregation, very efficient NIR emission arising from a ^3^MMLCT excited state with PLQY as high as 0.81 was obtained. As aforementioned, the perpendicular molecular arrangement, together with a highly ordered structure, allowed the exciton to diffuse over long distances with minimal vibrational relaxation to the ground state. Among these dopants, incorporation of **16** into an optimized planar non-doped OLED structure with architecture as follows ITO (100 nm)/1,4,5,8,9,11-hexaazatriphenylene hexacarbonitrile (HATCN) (10 nm)/NPB (50 nm)/mCP (15 nm)/**16** (20 nm)/2,2′,2′′-(1,3,5-benzenetriyl)-tris(1-phenyl-1*H*-benzimidazole) (TPBi) (60 nm)/8-hydroxyquinolatolithium (Liq) (2 nm)/Al (100 nm), led to an EQE of 24 ± 1%. This result was even improved when a light outcoupling hemisphere structure was employed, achieving outstanding values of EQE up to 55 ± 3%. This performance is the highest reported so far for a NIR OLEDs. Therefore, these works nicely showed how both crystallinity and molecular orientation are key parameters that can make great differences for the resulting thin-film optoelectronic performances.

**Figure 9:**
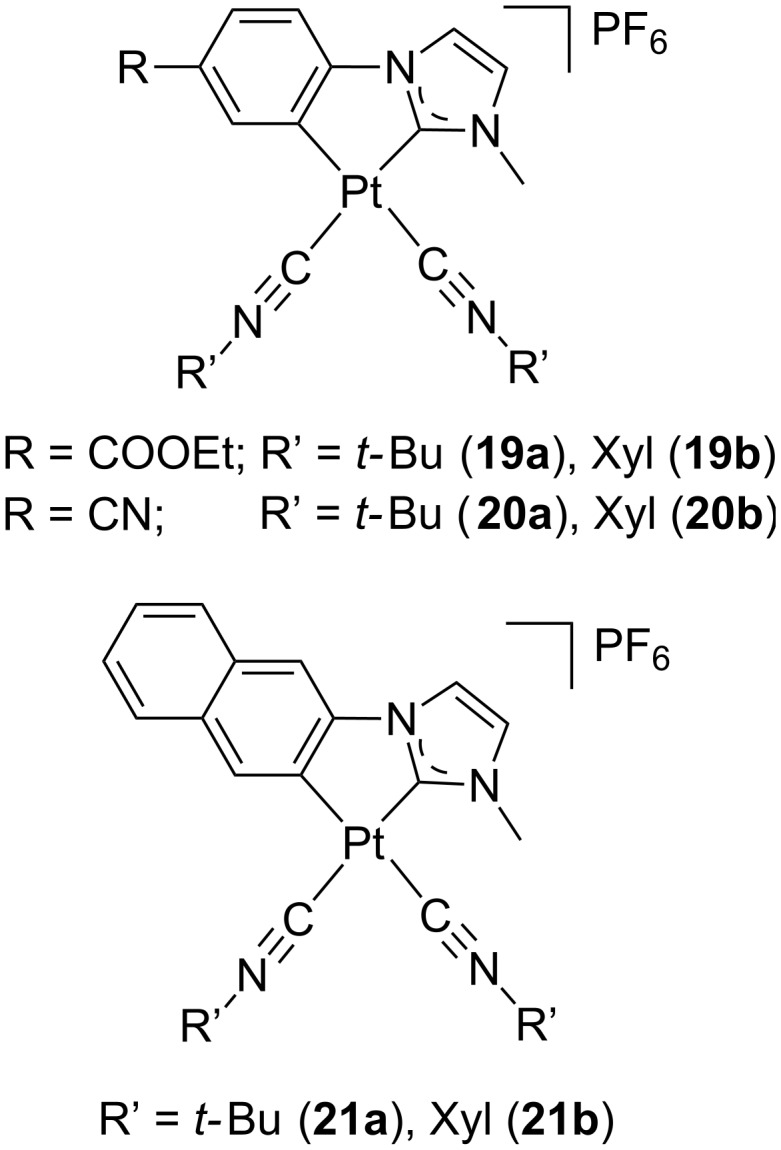
Molecular structure of carbene-containing cyclometallated alkynylplatinum(II) complexes **19**–**21** [41].

Apart from display applications, general lighting efficiency currently constitutes a main concern of our society and white-emitting OLEDs (WOLEDs) represent a valuable alternative because of their energy-saving potential. In this regard, development and improvement of white-light emitting devices attracts considerable interest. Nowadays, two main fabrication strategies seemed to be the most promising ones such as i) including either three (RGB) or two emitting components (sky-blue-orange); ii) using a phosphorescent material to partially down-convert UV or blue light from a LED source; the latter seems a promising option to date. The group of Sicilia has recently applied some cyclometallated platinum(II) complexes bearing NHC ligands to develop WOLEDs, whose chemical structure is sketched in Figure 9 [41]. Depending on the π-conjugation of the NHC-based bidentate ligand, emitting complexes with luminescence varying from blue (**19** and **20**) to yellow (**21**) were obtained. Several devices were prepared following a remote phosphor configuration, which places the phosphors spatially separated from the LED source. The associated values of correlated colour temperature (CCT), colour rendering index (CRI) and luminous efficacy of the radiation (LER) were acceptable, proving the suitability of these systems for lighting applications. Nevertheless, a fast degradation of the emission was observed under device operation.

#### Systems based on tridentate ligands

During the last two decades, platinum(II) complexes bearing tridentate ligands have been extensively investigated as well. Compared to their mono- and bidentate counterparts, a three-fold chelating motif imposes higher geometrical rigidity, which is expected to further decrease molecular distortions. The overall stability of the resulting compound is increased, thus helping to greatly suppress nonradiative deactivation pathways. Although 2,2’:6’,2”-terpyridines showed widespread use in coordination chemistry [42–43], the bite angle of such class of tridentate ligands is not ideal for a square-planar geometry, leading to longer bond lengths when compared with their bidentate congeners. As a consequence, ligand-field is reduced and the presence of low-lying d–d excited states provide easy access to nonradiative deactivation channels [25,44].

Nevertheless, the use of multidentate chromophoric ligands that are able to provide metal–ligand bonds with higher covalent character, as for instance cyclometalating ligands, has proven to be a successful strategy for improving the luminescence properties due to the energetic destabilization of quenching MC states [45–46].

#### Complexes based on C^N^N ligands

Following the seminal work of von Zelewski [47–48] on platinum(II) complexes bearing C-deprotonated 2-phenylpyridines (C^N), the development of tridentate analogues has received a great deal of attention in the recent past. Early reports were based on 6-phenyl-2,2’-bipyridine, namely C^N^N [49–50]. In spite of the strong ligand field exerted by the cyclometalating moiety, this type of complexes resulted to be rather weakly emissive due to large structural distortion of the emitting triplet excited state. Nevertheless, Che and co-workers demonstrated that extending the π-conjugation of the cyclometalated ligand led to enhanced phosphorescence quantum yields [51–52]. Indeed, the increased conjugation resulted in a modification of the frontier molecular orbitals and prevention of Jahn–Teller distortions.

**Figure 10:**
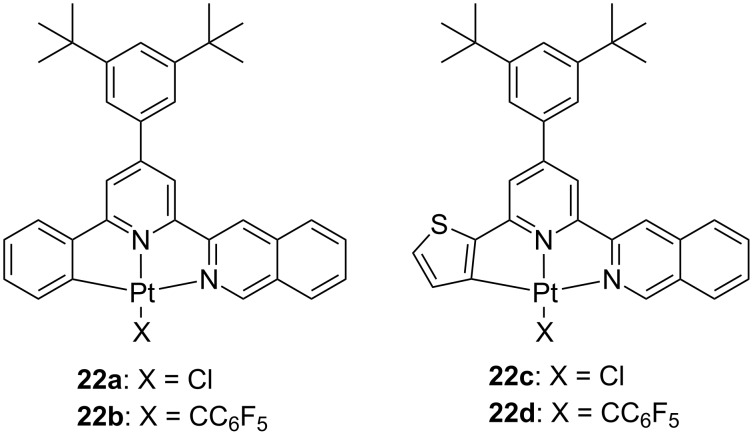
Chemical structure of platinum(II) complexes **22a–d** bearing asymmetric C^N^N tridentate ligands [53].

Recently, Che and co-workers reported a series of asymmetric tridentate C^N^N platinum(II) complexes with π-extended moieties, compounds **22** (Figure 10) [53]. Depending on the ancillary ligand, these complexes showed emission arising from several contributions, being ^3^MLCT and ^3^ILCT, together with ^3^XLCT or ^3^LLCT, where XLCT is a halogen-to-ligand charge transfer, with PLQY values approaching unity for some derivatives. Different structural isomers were synthesized, including a π-conjugated fragment attached at different positions of the employed tridentate ligand. The best results were obtained when the azine moiety isoquinolin-3-yl was used due to the minimization of the repulsions within the tridentate scaffold as well as with the ancillary ligands. Based on these initials results, new structural variations were investigated at both the cyclometalating and the ancillary ligands. As for the former, a clear impact on the emission colour was observed due to the participation of the cyclometalating unit to the HOMO frontier orbital. Thus, an emission ranging from green to yellow and finally to red was obtained going from phenyl, thiophene and benzothiophene cyclometalating rings, respectively. On the contrary, the ancillary ligand had a remarkable effect on the emission efficiency. In the case of pentafluorophenylacetylide, the change in the nature of the emitting excited state led to an almost negligible *k*_nr_ value, which resulted in an outstanding PLQY close to unity. The most promising complexes were selected by the authors as dopants for OLED fabrication and their chemical structure is displayed in Figure 10. Four devices with the configuration of ITO/TAPC (50 nm)/ TCTA:**22** (2–4 wt %, 10 nm)/TmPyPB (50 nm)/LiF (1 nm)/Al (100 nm) were fabricated, attaining CE of 23.1–76.7 cd A^−1^ and PE of 10.4–45.0 lm W^−1^. While devices fabricated with **22a**,**b** as dopant exhibited yellowish-green emission, those embedding **22c**,**d** showed saturated red colour. As for the maximum EQE, very high values up to 22.8% were achieved. These values are among the highest ones reported for platinum(II) complexes as dopant materials. It is worth to note that optimized PhOLED device embedding complex **22c** at doping concentration of 4 wt % showed an EQE of 22.1% that compares well with the best red-emitting iridium(III)-based devices.

#### Complexes based on N^C^N ligands

**Figure 11:**
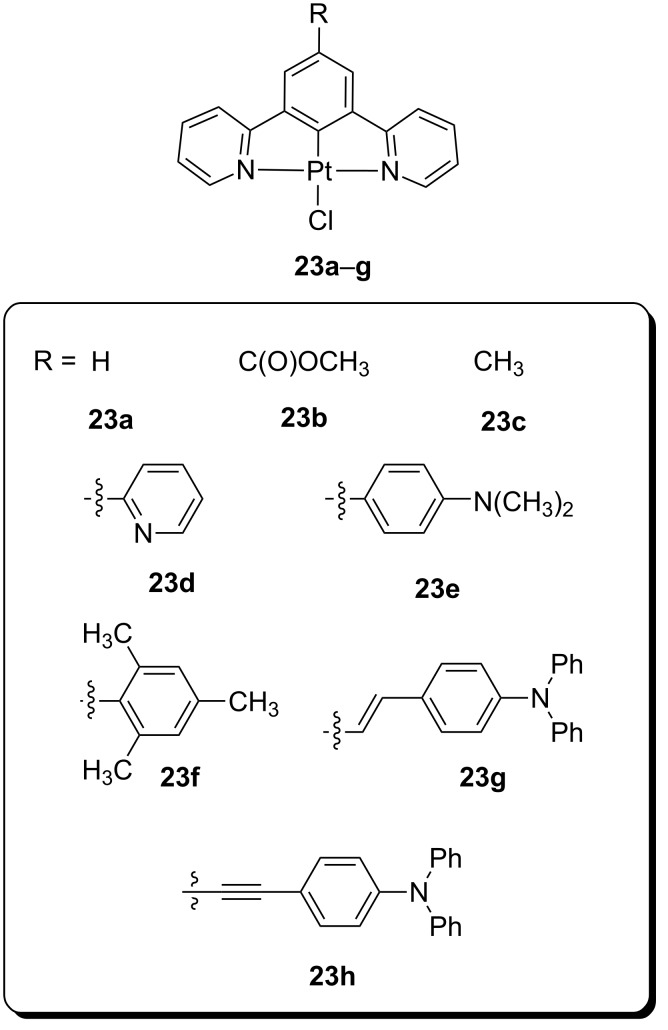
Chemical structure of platinum(II) complexes **23** bearing *bis*-cyclometalating 2,6-dipyridylbenzene type of ligands [54–56].

Although formally bearing similar coordinating units, platinum(II) complexes bearing symmetrical N^C^N ligands resulted in better emitters than those bearing the corresponding C^N^N motif. For instance, while [Pt(C^N^N)Cl] (C^N^N = 6-phenyl-2,2’-bipyridine) possess a rather low emission (PLQY = 0.025) in degassed CH_2_Cl_2_ solution at room temperature [50], [Pt(N^C^N)Cl], where N^C^N is a bis-cyclometalating 2,6-dipyridylbenzene type of ligand (complexes **23**), displays a much higher PLQY reaching 0.60 in similar conditions, as for instance compound **23a** [54]. The chemical structure of complexes **23** is shown in Figure 11. These distinct results can be interpreted as follows. A shorter Pt–C bond length was observed for the N^C^N-containing complex, revealing a stronger interaction with the metallic ion. As a consequence, a higher d–d splitting could be foreseen, thereby reducing the possibility of a non-radiative deactivation channel of the emitting excited-state. On the other hand, [Pt(N^C^N)Cl] displayed a metal-perturbed ^3^π–π* emission as also demonstrated by the relatively high radiative rate constant value. The combination of these two factors explained well the aforementioned good emission efficiencies. As a result, N^C^N-coordinated complexes have found numerous applications as emitting materials in areas such as emitters in PhOLEDs [55–56] and luminescent probes in bio-imaging [57–59]. Noteworthy, NIR-emitting OLED were fabricated by using complexes **23g** and **23h**, which presented a π-delocalized substituent at the 5-position of the central phenyl ring. As the parent complex **23a**, excimer formation via metal–metal interactions was observed for both derivatives at high concentrations or in neat films. Nevertheless, the increased conjugation within the chromophoric ligand led to a lower emission energy, which fell into the NIR region. The structure of the optimized vacuum-processed OLED was as follows: ITO (120 nm)/Mo_2_O_x_ (2 nm) / TCTA (80 nm) /**23g** or **23h** (15 nm)/TPBi (25 nm)/LiF (0.5 nm)/Al (100 nm). Complex **23g** attained remarkable performances for this class of Pt(II)-based compounds, with an EQE of 1.2% at a current density of 10 mA cm^−2^ and an electroluminescence intensity of about 10 mW cm^−2^ at 9 V.

**Figure 12:**
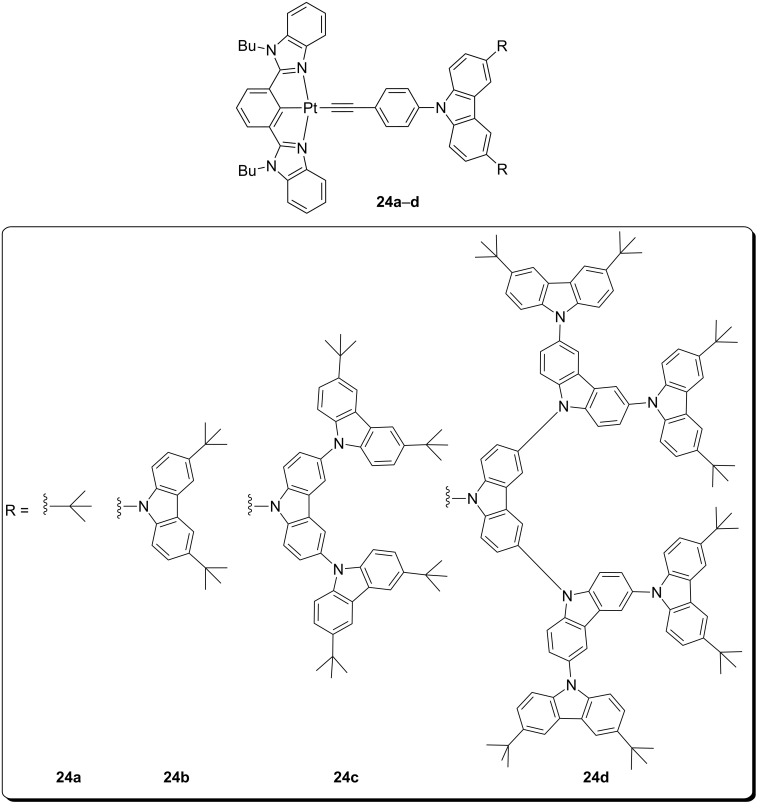
Molecular structure of dendritic carbazole-containing alkynyl-platinum(II) complexes **24a–d** [62].

Due to the triplet character of typical platinum(II) complex emission, these metal-based dopant phosphors are typically dispersed in high triplet energy hosts to suppress energy transfer processes onto the host matrix that detrimentally affect the final performances [60]. Alternatively, development of emissive complex incorporated in a dendritic structure allows controlling both charge transport and light emission in a single material [61]. In this regard, Yam and co-workers reported on a series of dendritic carbazole-based alkynylplatinum(II) complexes with cyclometalated 2,6-bis(*N*-alkylbenzimidazol-2’-yl)benzene (bzimb) as the N^C^N tridentate ligand [62]. These complexes were found to be highly emissive with PLQYs of up to 0.80 in solid-state thin films. Contrarily to other alkynylplatinum(II) complexes, their emission was ascribed to an admixture of ^3^IL/^3^MLCT since no influence of the dendrimeric ancillary ligand was observed. Nevertheless, upon increasing the dopant concentration in thin films up to 50 wt %, a new low-energy band was observed that was attributed to the formation of excimeric species. Nonetheless, it is worth to note that this excimeric emission was reduced on increasing the generation of the ancillary ligand, highlighting the importance of this molecular design strategy towards highly efficient dopants. The interesting photophysical properties of these compounds prompted the evaluation of their electroluminescence performances in OLED devices. Solution-processed green-emitting PhOLEDs were prepared with the structure of ITO/poly(ethylenedioxythiophene):poly(styrene sulfonic acid) (PEDOT:PSS 70 nm)/mCP:**24** 5–50 wt % (60 nm)/SPPO13 (30 nm)/LiF (0.8 nm)/Al (100 nm), where SPPO13 is 2,7-bis(diphenylphosphoryl)-9,9′-spirobifluorene. For all devices, emission similar to those recorded in solution was obtained independently of the doping concentration. Moreover, the decreasing driving voltages measured were ascribed to better charge transport properties in the emissive layer upon increasing the dendron generation. However, the best hole-electron current balance was achieved for a platinum(II) complex with the second generation dendrimeric structure (Figure 12), yielding a maximum CE and EQE of 37.6 cd A^−1^ and 10.4%, respectively. This enhanced performance highlights the beneficial effect of employing emitters with a dendrimeric design. Indeed, these results were among the best values ever reported for PhOLEDs based on metal-containing dendrimers, and even compared well with vacuum-deposited devices of non-dendritic structurally-related platinum(II) complexes.

**Figure 13:**
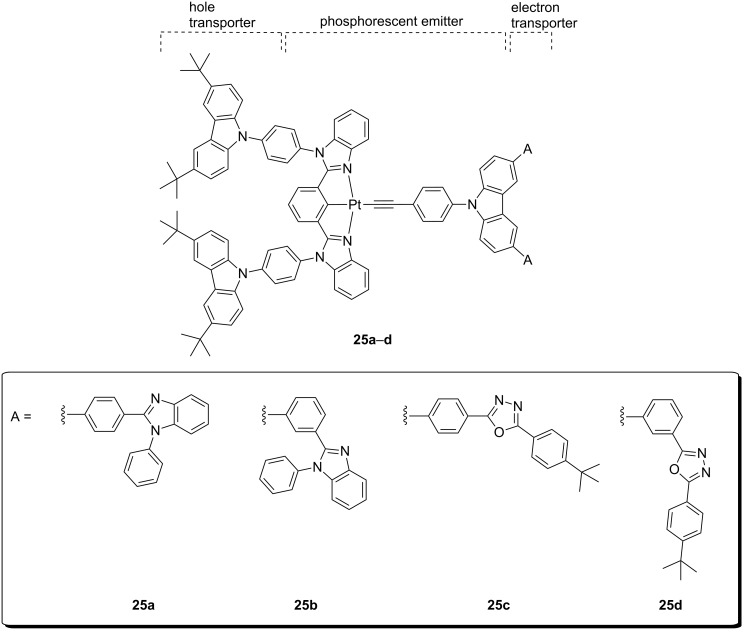
Molecular structure of bipolar alkynyl-platinum(II) complexes **25** bearing carbazole and electron-accepting phenylbenzimidazole or oxadiazole moities [63].

As a further development of the work, the same group reported very recently another family of platinum(II) complexes containing both electron-donor and electron-acceptor moieties embedded within the dopant structure (Figure 13) [63]. This bipolar character was intended to reduce the TTA phenomena commonly experienced at high current density that leads to severe roll-off efficiency of OLEDs [64]. In particular, carbazole-based donor moieties and either phenylbenzimidazole (PBI) or oxadiazole (OXD) accepting units were selected as the hole-transporting and electron-transporting moiety, respectively. Two linkage fashions were explored between these donor-acceptor groups, namely *meta*- and *para*-substitution. As expected, the intramolecular charge transfer character was less prominent in the absorption features of compounds with *meta*-linkages. Nevertheless, all compounds showed a ^3^IL/^3^MLCT emission in the green region, that resembled well that of other complexes bearing the bzimb tridentate ligand, with no influence of the connecting mode. Moreover, successful energy transfer was achieved upon doping thin films of TCTA:SPPO13 with the tridentate platinum complex, and high PLQY in the range 0.62–0.75 were achieved. These promising results prompted the authors to fabricate PhOLED devices employing these new bipolar emitters. The device architecture was as follows: ITO/PEDOT:PSS (70 nm)/**25**:TCTA:SPPO13 5–20 wt %:1:1 (60 nm)/1,3-bis(3,5-bis(pyridine-3-yl)phenyl)benzene (BmPyPhB; 30 nm)/LiF (0.8 nm)/Al (100 nm). The differences in molecular design became more evident under operational device conditions. The emitters with OXD units performed better than those with PBI units. On the other hand, a remarkable increase of CE and EQE was obtained going from *para-* to *meta-*linkage. As a result, CE as high as 57.4 cd A^−1^ were reached along with a EQE of 16.0%, for the *meta*-connected OXD-containing platinum(II) dopant at 15 wt %. These interesting results demonstrated the beneficial effects of bipolar metal-based emitters for high-performing optoelectronic devices.

**Figure 14:**
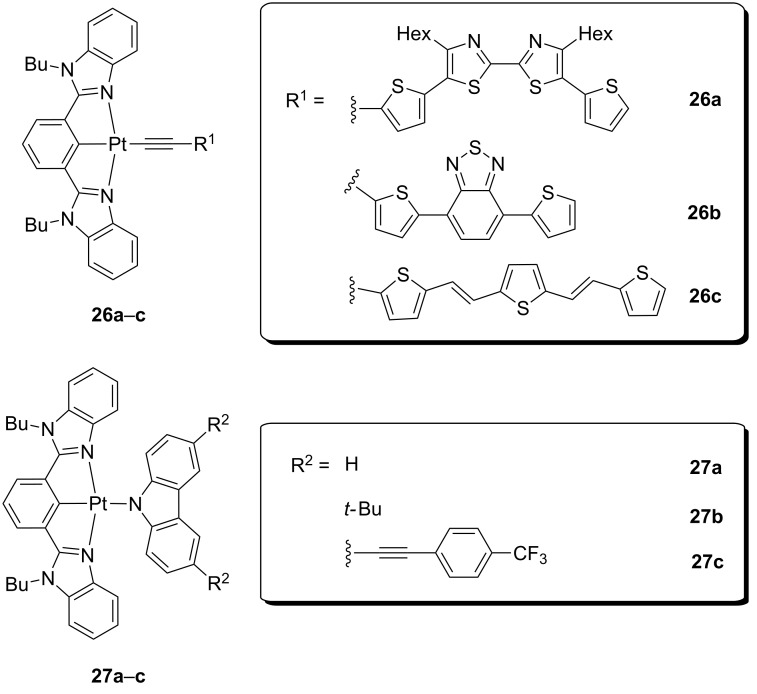
Molecular structures of neutral platinum(II) complexes comprising donor-acceptor alkynyls (**26**) or electron-rich carbazoles (**27**) as ancillary ligands [65].

In another study from the group of Yam, the bipolar design was conceived to finely tune the emission energies of the compounds [65]. Two series of platinum(II) alkynyl (compounds **26**) and carbazoyl (compounds **27**) complexes were reported, which included different donor and/or acceptor groups on the ancillary ligand (Figure 14). As expected, their emission behavior was strongly dependent on the nature of this latter, displaying different combinations of π–π* and charge-transfer triplet excited states, together with a broad emission ranging from the green to the red portion of the spectrum. Interestingly, a solution-processed OLED fabricated with a complex bearing a carbazoyl ancillary ligand showed concentration-dependent electroluminescence. In addition, a change in nature of the emission from ^3^IL to ^3^MLCT/^3^LLCT character was observed upon increasing doping concentration from 5 to 20 wt %. Moderate performances were attained at this latter concentration, with CE of 24.0 cd A^−1^ and EQE of 7.2%. Alternatively, these compounds were successfully employed in the fabrication of organic memories, which demonstrates the great versatility of this class of platinum(II)-containing materials.

#### Complexes based on bis-anionic C^N^C and N^N^N ligands

In an attempt to further destabilize the d–d excited states, doubly cyclometalating 2,6-diphenylpyridine [66–67] and their extended π-conjugated analogues have been employed as C^N^C tridentate ligands for platinum(II) complexes. Nevertheless, the resulting complexes resulted to be almost nonemissive in solution at room temperature in spite of the stronger ligand-field exerted. Similar to the case of C^N^N type of ligands, a significant structural distortion is the main factor that accounts for this low emission efficiency. However, Che and co-workers demonstrated that extension of the π-conjugation at the tridentate ligand, together with the use of heterocyclic moieties such as thiophene or carbazole, clearly favours the luminescence properties of these type of platinum(II) complexes [68].

**Figure 15:**
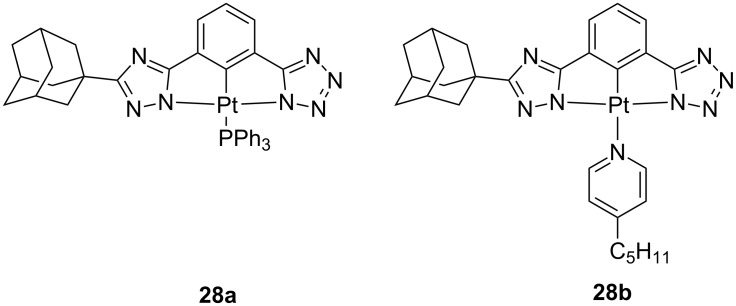
Chemical structure of the asymmetric Pt(II) derivatives **28** bearing triazole and tetrazole moieties onto a tridentate ligand [75].

As aforementioned, N-deprotonable azole units constitute a compelling alternative to C-cyclometalating ligands [16]. In this regard, dianionic tridentate N^N^N ligands bearing pyrazolate [69], triazolate [69–73] or tetrazolate [74] units have been used to successfully prepare highly luminescent neutral platinum(II) complexes in dilute solution and/or as aggregated state. Due to their promising emitting features, these complexes have also been employed as phosphors in optoelectronic devices [71–72]. Neutral platinum(II) complexes with an asymmetrical triazolate- and tetrazolate-containing tridentate ligand, complexes **28**, were also reported [75] (Figure 15). These green emitters were used to fabricate solution-processed PhOLEDs, displaying performances as high as their vacuum-processed structurally-related analogues, with a maximum PE of 16.4 lm W^−1^, CE of 15.5 cd A^−1^ and EQE of 5.6% obtained for derivative **28b**. These performances are amongst the highest EQE values for solution-processed platinum-based OLEDs.

#### Systems based on tetradentate ligands

Tetradentate ligands have attracted an increased attention due to the even higher rigidity of the chromophoric scaffold that helps to suppress nonradiative decay pathways induced by large distortions around the metal atom [76–77].

**Figure 16:**
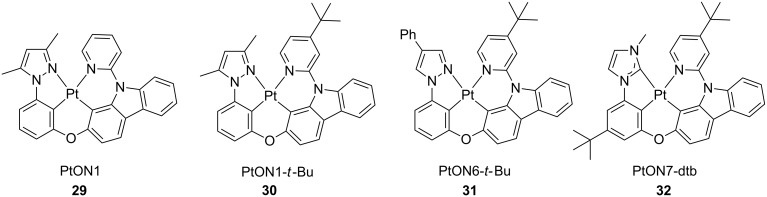
Molecular structure of the tetradentate platinum complexes **29**–**32** bearing N^C^C^N and C^C^C^N ligands [79].

Following on their strategy of employing rigid N^C^C^N and C^C^C^N ligands bearing either methyl-2-phenylimidazole or phenylpyrazole moieties [78], Li and co-workers recently reported on a series of tetradendate platinum(II) complexes **29**–**32** that displayed narrow emission spectral bandwidth (Figure 16) [79]. In such derivatives, the introduction of an electron-donating moiety, such as a *tert*-butyl group, onto the pyridyl ring of the tetradentate scaffold induces a larger energy separation between the carbazolepyridine and the phenylpyrazolate moieties. In consequence, spectra are narrowing and a higher colour purity can be achieved by reducing vibronic sideband contributions to the overall emission spectrum. The complexes displayed PLQY above 0.7 in PMMA thin-film with λ_em_ maxima centred at ca. 450 nm. OLED devices employing complexes **30**–**32** as emitting materials were fabricated with the following architecture: ITO/HATCN (10 nm)/NPD (40 nm)/TAPC (10 nm)/Pt complex 2 wt %: 26mCPy (25 nm)/DPPS (10 nm)/BmPyPB (40 nm)/LiF/Al, where 26mCPy, DPPS are 2,6-bis(*N*-carbazolyl)pyridine and diphenyl-bis[4-(pyridin-3-yl)phenyl]silane, respectively. All the investigated derivatives showed an EL spectrum similar to the PL emission band indicating efficient suppression of the spectral broadening thanks to the bulky *tert*-butyl groups. Thus, “pure” blue electroluminescence with CIE_y_ coordinate <0.1 and EQE of 17.2% were achieved for derivative **32** bearing a NHC ligand. Interestingly, upon increasing doping concentration from 2 to 6 wt % and employing TAPC and a higher bandgap electron transporting material 2,8-bis(diphenylphosphoryl)dibenzothiophene (PO15) at 1:1 ratio as co-host, peak EQE of 24.8% was achieved without significantly affecting colour purity.

**Figure 17:**
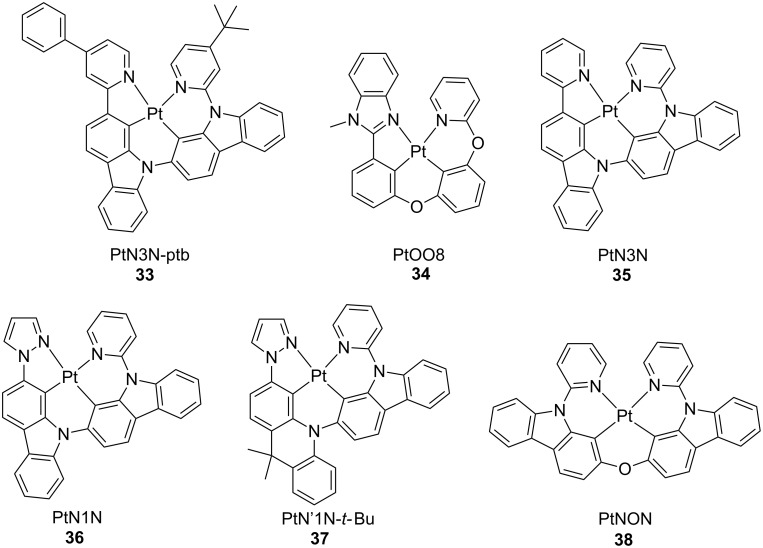
Chemical structure of the tetradentate Pt complexes **33**–**38** based on N^C^C^N-type of ligands [80–84].

Variation of the emissive moiety from the methylimidazole or phenylpyrazole to the 4-phenylpyridyl carbazole afforded compound **33** (Figure 17). This complex displayed an emission maximum at 602 nm in CH_2_Cl_2_ arising from an excited state with strong ^3^MLCT character with PLQY of 0.34 (Figure 17) [80]. OLEDs were fabricated with device architecture as follows: ITO/HATCN(10 nm)/NPD(40 nm)/TrisPCz (10 nm)/**33** 10 wt %:CBP(25 nm)/BAlq(10 nm)/BPyTP(40 nm)/LiF(1 nm)/Al(100 nm), where TrisPCz, CBP and BAlq is 9,9′,9″-triphenyl-9*H*,9′*H*,9″*H*-3,3′:6′3″-tercarbazole, 4,4**′**-bis(*N*-carbazolyl)biphenyl and bis(2-methyl-8-quinolinolato)(biphenyl-4-olato)aluminum, respectively. The devices showed orange-red electroluminescence with remarkable estimated 97% operational lifetime, LT_97_, over 600 hours at 1000 cd cm^−2^ and peak EQE of 10.8%. Nonetheless, further improvement of the device efficiency upon variation of host material increased the EQE value up to 21.5% when a dopant concentration of 2 wt % and the ambipolar Bebq2 host were employed instead, where Bebq2 is bis(benzo[*h*]quinolin-10-olato-κN,κO)beryllium(II). In spite of that, much lower LT_97_ values were observed most likely due to a higher charge and exciton concentration in the host layer at such low doping concentration. Such compound represents the most stable Pt(II) complex used as emissive material in an OLED device to date.

Compound **33** together with **34** and **29** were subsequently employed by the same authors as red, green and blue emissive materials, respectively, for the fabrication of white-light OLEDs (WOLEDs) [81]. Upon optimization of the device architecture in terms of doping concentration, layer thickness and stacking order of each of the emissive materials, WOLED devices with the following architecture ITO/HATCN/NPD/TAPC/complex **33** 6 wt %:26mCPy (3 nm)/complex **29** 6 wt %:26mCPy (20 nm)/complex **34** 6 wt %:26mCPy (2.5 nm)/DPPS/BmPyPB/LiF/Al showed CIE (x, y) coordinates of 0.35, 0.35, CRI of 80 and maximum EQE of 21.0%. However, a large efficiency roll-off was observed at higher current density due to increased charge and exciton trapping.

Further modification of the structure of complex **33** resulted in the related compound **35** that showed a more intense (PLQY = 0.63) and orange-red emission band with the maximum centered at 582 nm and an excited state lifetime of 7.3 μs in CH_2_Cl_2_ at room temperature [82]. EL performances were investigated in a charge balanced OLED device, with bi-layer EML architecture comprising two different dopant concentrations in order to shift exciton formation zone deeper into the emissive layer (device configuration: ITO/HATCN/NPD/TrisPCz/compound **35** 20 wt %:CBP/compound **35** 6 wt %:CBP/BAlq/BPyTP/LiF/Al). Such devices displayed EL spectra that was slightly broader than PL emission due to the relatively high dopant concentration with an estimated LT_97_ = 2057 h and EQE = 15.3% at 1000 cd m^−2^.

Seeking for stable and efficient blue emitter for OLED devices and following the previous work on the red-emissive compound **33** and the green-emissive derivative **36** that showed a peak EQE of 14.3% [83], Li and co-workers developed a novel blue-emitting tetradentate platinum complex, namely **37**. The excited state of this compound was raised by breaking the π-conjugation of the carbazole moiety upon introduction of 9,10-dihydro-9,9-dimethylacridine moiety, where the two methyl groups were introduced to minimize oxidation of the benzyl carbon under device operation (Figure 17) [84]. Compound **37** exhibited a maximum of emission at 486 nm with a spectrum characterized by vibronic features, most likely due to the increased flexibility of the acridine moiety that imparted a more distorted excited state geometry compared to the carbazole-based counterpart. Upon device optimization, **37** resulted to be a rather efficient sky-blue triplet emitter. In particular, OLEDs with the following architecture ITO/HATCN (10 nm)/NPD (40 nm)/TrisPCz (10 nm)/complex **37** 10 wt %:mCBP (25 nm)/mCBT (8 nm)/BPyTP (40 nm)/LiF (1 nm)/Al (100 nm) were fabricated that showed peak EQE = 17.8% and LT_70_ of 482 h at 1000 cd m^-2^.

A similar strategy based on the rupture of the π-conjugation in a cyclometalating ligand was employed by the same authors to achieve blue emission in symmetric tetradentate platinum(II) complexes **38** bearing six-membered pyridyne-carbazole chelating rings [85]. This latter compound showed modest (PLQY = 0.31) photoluminescence peaking at 508 nm in CH_2_Cl_2_ solution at room temperature. Interesting, drop-casted PMMA thin-film prepared at 5 wt % doping level exhibited hypsochromically shifted emission (λ_em_ = 474 nm) with much higher intensity (PLQY = 0.83) making such compound a valuable candidate for blue-emitting OLEDs. Upon embedding compound **38** at 6 wt % doping level in a charge and exciton confining structures with the following architecture ITO/HATCN (10 nm)/NPD (40 nm)/TAPC(10 nm)/complex **38**: 26mCPy (25 nm)/DPPS (10 nm)/BmPyPB (40 nm)/LiF/Al, OLED devices with peak EQE of 24.4% were fabricated. Such efficiency is comparable to the best blue iridium and platinum complexes reported so far.

**Figure 18:**
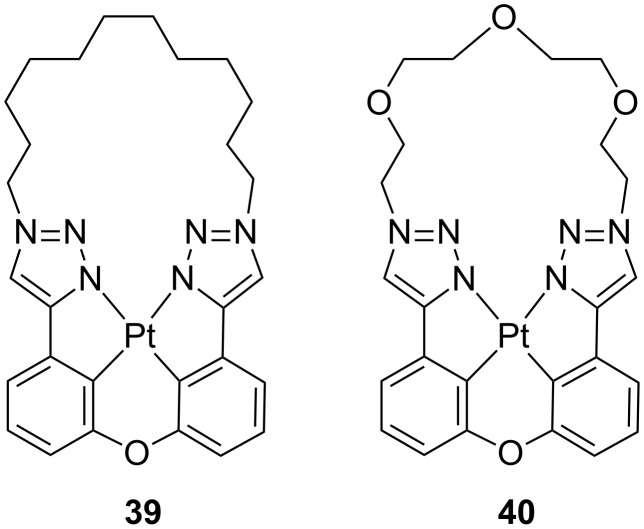
Chemical structure of the macrocyclic tetradentate platinum complexes reported by Wang and co-workers [86].

Two different classes of tetradentate platinum derivatives bearing N^C^C^N rigid ligands were recently reported by Wang and co-workers, bearing either bis(1,2,3-triazolylphenyl) [86] or bis(1,2,4-triazolylphenyl) ligands [87]. Examples of the former class, namely complexes **39** and **40**, are displayed in Figure 18. In particular, these complexes were designed to reduce excited-state distortions by bearing a macrocyclic chelating ligand and either ether, methylene or carbonyl bridging units. The derivatives showed bright blue phosphorescence centred at λ_em_ ca. 448–470 nm depending on the bridging unit. Such blue emission was retained when the complexes were embedded in PMMA rigid matrix. Interestingly, macrocyclic derivatives possessed higher PLQY in solution with values of 0.58–0.62 when compared to non-macrocyclic counterparts that was attributed to the enhanced structural rigidity imposed by the cyclic structure. By employing complex **39** as emissive material OLED devices with the following architecture were fabricated: ITO/NPB (50 nm)/mCP (10 nm)/9,9′-(4,4′-(phenylphosphoryl)bis(4,1-phenylene))bis(9*H*-carbazole) (BCPO):complex **39** *x* wt % (20 nm)/bis[2-(diphenylphosphino)phenyl] ether oxide (DPEPO) (10 nm)/TPBi (30 nm)/LiF (1 nm)/Al (100 nm) with doping level *x* of 2, 5 and 10 wt %. EL spectra showed an emission peak at λ_EL_ = 452 nm that did not show any dependency on the doping concentration and a rather low turn-on voltage of 3.2 V. The best EL performances were recorded for the OLED device at 10 wt % doping level that showed peak brightness, CE and PE of 10680 cd m^−2^, 11 cd A^−1^ and 10.8 lm W^−1^, respectively, and EQE value of 9.7%. In a second set of deep-blue OLED devices, maximum EQE of 15.4% were achieved at brightness of 490 cd m^−2^.

**Figure 19:**
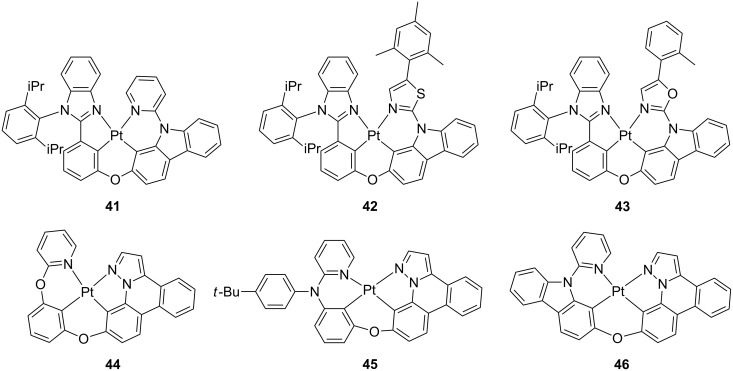
Molecular structure of complex **41–46** [88–89].

Other classes of tetradendate platinum(II) complexes bearing N^C^C^N chromophoric ligands have been recently reported by Fan and coworkers [88–89]. In order to prevent detrimental intermolecular interactions which might largely affect colour purity and emission efficiency in a condensed state, as well as increase solubility of the complex, the authors developed a series of (2-phenylbenzimidazole)-based tetradentate Pt(II) complexes bearing a diisopropylphenyl group, which is orthogonally oriented with respect to the molecular plane [88]. The three complexes featured 2-pyridylcarbazole (**41**), 2-thiazolylcarbazole (**42**) and 2-oxazolylcarbazole (**43**) moieties employed as the luminophoric motifs that were linked to the 2-phenylbenzimidazole unit through an ether bridge (Figure 19). The three complexes exhibited high thermal stability since thermogravimetric analysis (TGA) showed a weight loss of only 5% at temperatures in the range 436–463 °C. An intense and structured emission in the green region with λ_em_ = 500–507 nm and PLQY = 0.6–0.78 was recorded when the complexes were used as dopant in PMMA thin-film. DFT calculations helped to ascribe the nature of the frontier molecular orbitals as being carbazole/phenoxy and phenylbenzimidazole for HOMO and LUMO, respectively.

OLED devices were fabricated employing complexes **41**–**43** as emitting dopants with the following architecture ITO/HATCN (10 nm)/TAPC (40 nm)/TCTA (10 nm)/26mCPy:complex **41**–**43** *x* wt % (20 nm)/TmPyPB (45 nm)/Liq (2 nm)/Al (120 nm) with doping level *x* of 8, 10, 15 and 20 wt %. Even for the highest doping level investigated, i.e., 20 wt %, the EL emission was similar to the PL spectra observed in dilute condition, which suggests that the steric hindrance imparted by the diisopropylphenyl group is important for avoiding intermolecular interactions. Furthermore, OLED using complex **41** as emitting materials showed good performances with maximum EQE of 22.3%.

In a following study, a second series of tetradentate platinum complexes bearing a pyrazolo[1,5-*f*]phenanthridine moiety and with a general coordination motifs of the type N_pyridine_^C_phenyl_^C_phenyl_^N_pyrazole_ was reported by the same group, namely complexes **44–46** (Figure 19) [89]. The complexes showed moderate to intense sky-blue emission with PLQY in the range 0.2–0.7 and high thermal stability. Unfortunately, going from dilute solution to neat solid-state samples, PLQY values dramatically dropped to values as low as 0.10–0.02 that might point to strong intermolecular interaction and TTA phenomena. The tendency toward aggregation for complex **44** and **46** in condensed phase was also evidenced in the EL spectra. Although its shape was independent from the doping ratio, a bathochromically shift was observed along with a featureless emission profile. In sharp contrast, compound **45** displayed an EL emission maximum similar to that observed for the solution sample, indicating a much less pronounced aggregation. OLED devices were fabricated with the following configuration comprising different doping level: ITO/HATCN (10 nm)/TAPC (45 nm)/TCTA (10 nm)/host material:complex **44**–**46** *x* wt % (20 nm)/TmPyPB (50 nm)/Liq (2 nm)/Al (110 nm), where host was CBP for **44** and **46** and 26mCPy for compound **45**. Devices based on **44** at doping ratio as high as 30 wt % achieved the highest EL efficiencies amongst the three investigated complexes with CE, PE and EQE of 58.0 cd A^−1^, 51.6 lm W^−1^ and 16.4%, respectively.

**Figure 20:**
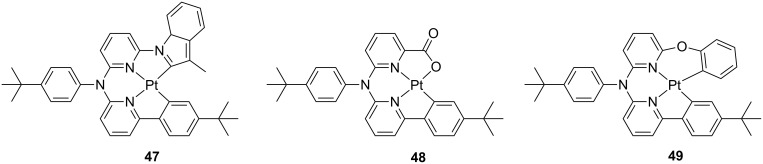
Molecular structure of asymmetric derivatives **47**–**49** based on triaryl-type of bridge [90].

The same authors have recently reported on another class of asymmetric [90] platinum complexes featuring tertiary arylamine motifs and their chemical structure is displayed in Figure 20. Such complexes, whose structure is derived from the parental symmetric systems previously reported by Huo and co-workers [91], bear a 3-methylindole, a carboxylic and a dangling phenoxy moiety, complex **47**, **48** and **49**, respectively, resulting in a general ligand structure with general formula being either C^N^N^C or C^N^N^O.

The compounds displayed moderate emission in the green-yellow portion of the visible spectrum with λ_em_ maximum peaking at 504–513 nm and PLQY of 0.27–0.47, attributable to an excited state with main LC character as suggested by the vibronic profile of the spectrum, repectively. Employment of these complexes as triplet emitters in OLEDs with configuration ITO/HATCN (10 nm)/TAPC (40 nm)/TCTA/mCP: platinum complex 10 wt %/TmPyPb (40 nm)/Liq (2 nm)/Al (120 nm) afforded electroluminescent devices with peak EQE of 13.3% and 13.6% for **48** and **49**, respectively. Even a higher peak EQE value of 16.3% was achieved for devices fabricated with **47** at similar doping level, although colour purity of the device resulted to be affected due to the fact that the EL emission resembles the PL spectra recorded in doped PMMA thin films rather than solution sample. This spectral broadening and shift is most likely due to the establishment of intermolecular interactions at such high doping level.

**Figure 21:**
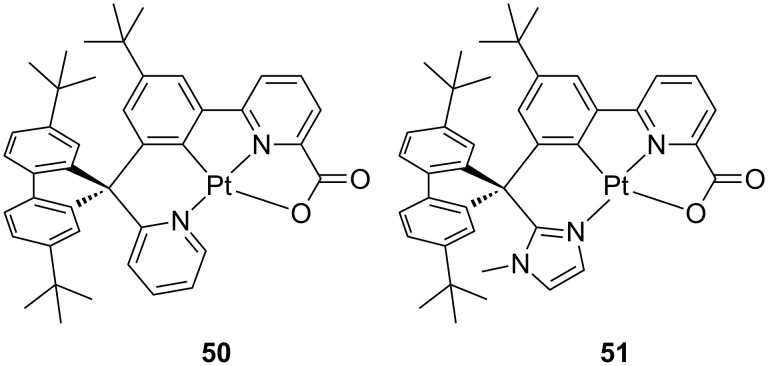
Chemical structure of the asymmetric tetradentate derivatives **50** and **51** based on spirofluorene linkage [96].

Indeed, platinum(II) complexes are well known to show both ground state aggregation phenomena including formation of metallophilic d^8^···d^8^ interactions and/or π–π stacking of the coordinating ligands [67,92] as well as excited-state interactions such as formation of excimers [93–94]. Although they may be usefully employed to shift both absorption and emission spectra, obtain long-range ordered luminescent supramolecular architectures and fabricate white-light emitting devices, aggregation phenomena of luminophors is typically considered detrimental due to the TTA and aggregation cause quenching (ACQ) processes that might take place. Thus, several strategies have been employed to date to avoid platinum emitters in close proximity, including introduction of bulky groups such as adamantyl [71] and spiro moieties [95]. By introducing on N^C^N^O tetradentate motifs both *tert*-butyl and spiro groups, Fan and co-workers recently reported on two platinum complexes, **50**–**51**, bearing a phenylpicolinate moiety. Their chemical structure is sketched in Figure 21 [96]. The complexes displayed structured luminescence with moderate PLQY (ca. 0.2) and relatively long lived-excited state lifetime in the range 8.4–11.6 μs. It is worth to notice that the presence of several bulky groups successfully suppressed aggregation as demonstrated by the similar PL spectra recorded in dilute CH_2_Cl_2_ and solid-state samples. Upon host material and doping ratio optimization, OLED devices achieved maximum EQE of 22.9% for complex **50** with relatively low roll-off efficiency that is attributed to the reduced quenching processes at high current density imparted by the bulky groups.

**Figure 22:**
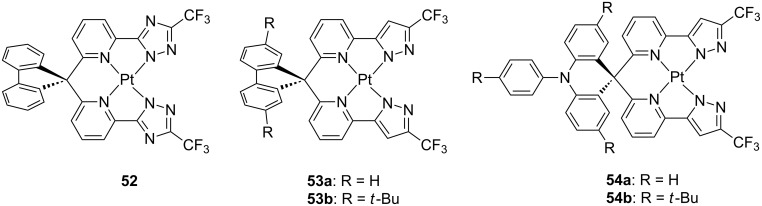
Molecular structure of the pyridylazolate-based complexes **52**–**54** reported by Chi and co-workers [97].

Spirofluorene and spiroacridine groups were also employed by Chi and co-workers on azolate-based tetradendate platinum complexes bearing either N_trz_^N_py_^N_py_^N_trz_ (**52**) and N_pz_^N_py_^N_py_^N_pz_ type (**53** and **54**) of ligands where trz and pz and py is a trifluoromethyltriazolate, trifluoromethylpyrazolate and pyridine ring, respectively [97] (Figure 22). This strategy has proven to enhance solubility and processability during device fabrication as demonstrated for a related Os(II) compound [98].

Photophysical characterization showed that complexes **52**, **53** and **54a** exhibited a structured and intense (PLQY = 0.58–0.8) blue emission with emission maxima at 452–465 nm. Complex **54b** was characterized by a large solvatochromic effect as a consequence of the large variation of the transition dipole moment from S_0_ to T_1_ states of 29.33 D. Indeed, while a structured phosphorescence ascribed to a ^3^LC/^3^MLCT transition has been observed in cyclohexane, a much broader and featureless profile is recorded in CH_2_Cl_2_ and ethanol, which underlies involvement of an emitting excited state with sizeable ILCT character becoming stabilized in such more polar solvents. The two derivatives displaying the highest PLQY among the series, namely **53b** and **54b**, were employed as triplet emitters in OLED device with architecture comprising an enlarged carrier recombination zone, such as ITO/TAPC (40 nm)/mCP:platinum complex 8 wt % (17 nm)/DPEPO platinum complex 8 wt % (3 nm)/TmPyPB (50 nm)/LiF (0.8 nm)/Al (150 nm). Devices fabricated with complex **54b** showed the highest peak efficiency of 15.3% with lower roll-off that was attributed to the better charge transport ability of compound **54b**. Furthermore, by combination of sky-blue emitter **53b** and **54b** and a red emitting osmium complex reported elsewhere [99], WOLED with a sandwiched recombination zone blue/red/blue emitters displayed warm-white emission with peak EQE of 12.7, CRI of 64 and CIE coordinate of 0.365, 0.376 at 1000 cd m^−2^.

**Figure 23:**
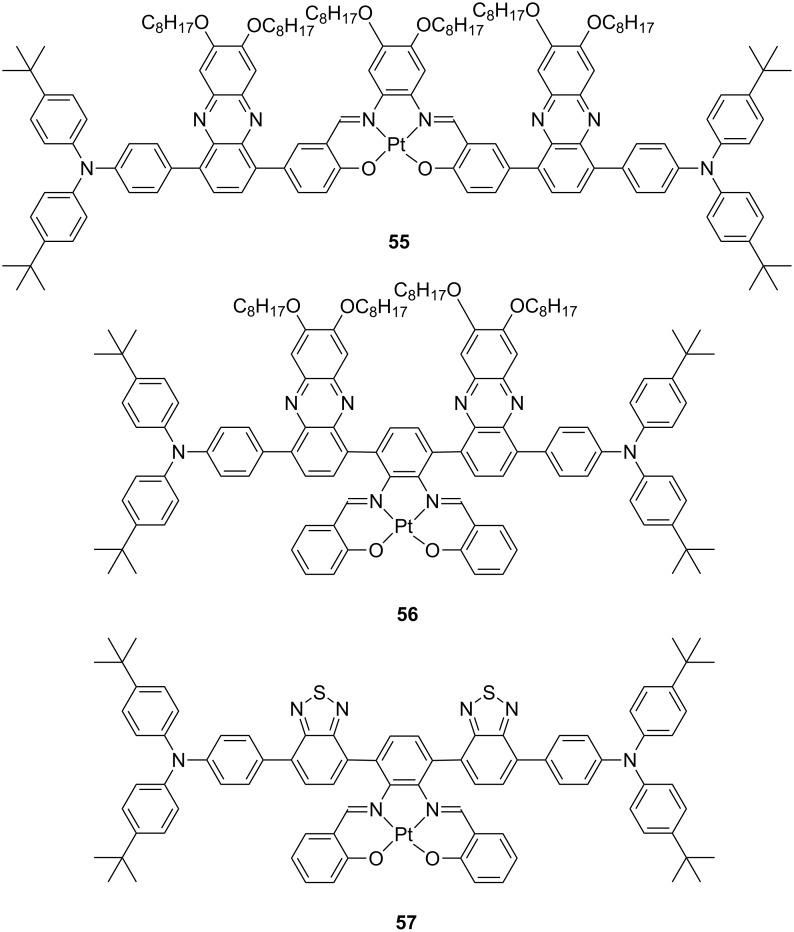
Chemical structure of the red-to-NIR emitting complexes **55**–**57** bearing donor–acceptor triphenylaminophenazine and triphenylaminobenzothiadiazole moieties [101–102].

Achieving efficient electroluminescence into the deep red and NIR region represents a challenging research topic of current interest, and only few examples are reported up to now showing remarkable performances [14]. Such challenge mainly arises from the intrinsic increase of the nonradiative rate constant upon decreasing the energy gap between excited and ground state that follows an exponential law known as energy gap law (EGL) [100]. In this respect, Su, Zhu and co-workers reported on two series of salophen-based tetradentate platinum(II) complexes decorated with donor–acceptor moieties such as triphenylaminophenazine [101] and triphenylaminobenzothiadiazole [102] and their chemical structure is shown in Figure 23.

All the complexes displayed long-lived red-to-NIR emissions in both solution and solid-state samples. The deepest red maximum was recorded for complex **57** with a maximum centred at λ_em_ = 697 nm arising from a triplet excited state with admixed MLCT/ILCT character as a consequence of the large donor–acceptor character of the ligand [102]. By employing complex **57** as triplet emitter in solution-processed OLED featuring a single-emissive layer, devices with architecture ITO/PEDOT (40 nm)/PVK:OXD-7:Pt complex 1–4 wt % (50 nm)/TPBI (30 nm)/Ba (4 nm)/Al (100 nm) were fabricated showing emission maximum λ_EL_ = 703 nm and peak EQE of 0.88% with relatively low roll-off efficiency upon increasing current density.

### Platinum(IV) complexes

**Figure 24:**
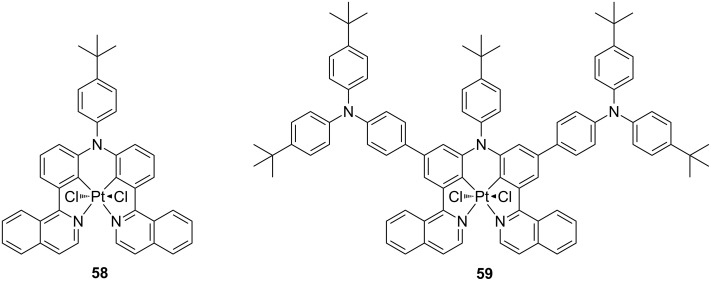
Molecular structures of the Pt(IV) derivatives **58** and **59** employed as triplet emitters in solution-processed OLEDs [110].

The first examples of luminescent platinum compounds with +IV oxidation states were reported by Balzani and von Zelewski back in the late 80s [103]. The complexes contained bis-cyclometalating (C^N) ligands of the general formula Pt(C^N)_2_(CH_2_Cl)Cl and were prepared by a photooxidative addition of CH_2_Cl_2_ onto the corresponding bis-cyclometalated Pt(II) parental complexes. Although Pt(IV) complexes have attracted great attention in cancer therapy [104–106], only in the very recent past they are receiving increasing interest as luminescent compounds [107–108]. Such derivatives are characterized by long-lived triplet-manifold π–π* excited states with either ^3^LC or ^3^ILCT nature. Most of the so far reported examples of octahedral Pt(IV) derivatives are based on heteroleptic and homoleptic systems containing phenyl-pyridine-type cyclometalating (C^N) ligands, reaching PLQY up to ca. 0.80 [109]. To date, only two examples of Pt(IV) derivatives, namely **58** and **59**, have been reported to be employed as active compounds in polymer-based OLEDs and their chemical structure is reported in Figure 24 [110]. The compounds contain a bis-cyclometalating tetradentate ligand scaffold based on phenyl-isoquinoline moiety decorated with hole-transporting triphenylamine groups, and two chlorine ancillary ligands in *trans* geometry. The complexes showed NIR luminescence (λ_em_ ca*.* 750 nm) in dilute 2-methyltetrahydrofuran solution and long-lived excited states with lifetime in the order of 0.7 μs.

To explore the potentiality of such phosphorescent Pt(IV) compounds as active materials in electroluminescent devices, solution-processed OLEDs with the following architecture ITO/PEDOT (40 nm)/PVK:complex (50–60 nm)/TPBi (30 nm)/Ba (4 nm)/Al, where PVK is poly(9-vinylcarbazole), were fabricated with dopant concentration adjusted in the range 1–8 wt % and their EL performances investigated. The devices showed interesting NIR emission similar to the PL spectra with emission maximum at λ_EL_ of about 750 nm for both compounds. Maximum radiant intensity and EQE of 164 μW cm^−2^ and 0.85% were recorded for compound **59** with relatively low roll-off at higher current densities.

**Table 1:** EL device performances reported for selected examples of luminescent platinum(II) and platinum(IV) complexes reviewed in this manuscript.

cmpd	device architecture	EL performances^a^				Ref. #

		CE (cd A^−1^)	PE (lm W^−1^)	Brightness (cd m^−2^)	EQE (%)	

**1**	ITO/TAPC (30 nm)/TCTA (10 nm)/DPEPO:**1** 10 wt % (20 nm)/DPEPO (10 nm)/TPBi (30 nm)/LiF/Al	–	–	–	8	[19]
**4**	ITO/TAPC (40 nm)/CBP:**4** 8 wt % (30 nm)/BP4mPy (40 nm)/LiF (0.8 nm)/Al (150 nm)	23.2	22.8	10 318	11.5	[26]
**8**	ITO/MoO_3_ (2 nm)/NPB (25 nm)/mCP (8 nm)/**8** neat (40 nm)/3TPyMB (50 nm)/LiF (1 nm)/Al	21.0	15.5	43 000	19.0	[27]
**10**	ITO/MoO_3_ (1 nm)/TAPC (65 nm)/mCP (8 nm)/**10** neat (30 nm)/3TPYMB (50 nm)/LiF (1 nm)/Al (120 nm)	90.0^b^	–	–	25.9^b^	[34]
**11a**	ITO/TAPC:MoO_3_ 20 wt % (20 nm)/TAPC (40 nm)/26DCzppy:**11a** 8 wt % (20 nm)/TmPyPB (50 nm)/LiF (0.8 nm)/Al (150 nm)	44.0	28.0	–	12.5	[35]
**13**	ITO (100 nm)/TAPC (80 nm)/ TCTA (10 nm)/**13** neat (30 nm)/BMPYPB (15 nm)/BMPYPB:Rb_2_CO_3_ 1 wt % (40 nm)/Al (100 nm)	62.0	53.8^c^	–	38.8	[13]
**16**	ITO (100 nm)/HATCN (10 nm)/NPB (50 nm)/mCP (15 nm)/**16** neat (20 nm)/TPBi (60 nm)/Liq (2 nm)/Al (100 nm)	–	–	–	24.0 (55)^d^	[14]
**22b**	ITO/TAPC (50 nm)/TCTA:**22b** 2 wt % (10 nm)/TmPyPB (50 nm)/LiF (1 nm)/Al (100 nm)	76.7	33.8	–	22.8	[53]
**22c**	ITO/TAPC (50 nm)/TCTA:**22c** 4 wt % (10 nm)/TmPyPB (50 nm)/LiF (1 nm)/Al (100 nm)	34.8	18.2	–	22.1	[53]
**23g**	ITO (120 nm)/Mo_2_O_x_ (2 nm)/TCTA (80 nm)/**23g** (15 nm)/TPBi (25 nm)/LiF (0.5 nm)/Al (100 nm)				1.2^e^	[56]
**24c**	ITO/PEDOT:PSS (70 nm)/mCP:**24c** 10 wt % (60 nm)/SPPO13 (30 nm)/LiF (0.8 nm)/Al (100 nm)	37.6	11.4	–	10.4	[62]
**25b**	ITO/PEDOT:PSS (70 nm)/TCTA:SPPO13:**25b** 1:1:15 wt % (60 nm)/BmPyPhB 30 nm)/LiF (0.8 nm)/Al (100 nm)	57.4	–	–	16.0	[63]
**28b**	ITO/PEDOT:PSS (32 nm)/QUPD (10 nm)/OTPD (8 nm)/PVK:OXD-7:**28b** 13.5 wt % (30 nm)/TPBi (25 nm)/CsF (3 nm)/Al (120 nm)	15.5	16.4	–	5.6	[75]
**32**	ITO/HATCN (10 nm)/NPD (40 nm)/TAPC (10 nm)/TAPC:PO15:**32** 47 wt %:47 wt %:6 wt % (25 nm)/PO15 (10 nm)/BmPyPB (30 nm)/LiF/Al	–	–	–	24.8	[79]
**33**	ITO/HATCN(10 nm)/NPD(40 nm)/TrisPCz(10 nm)/Bebq2:**33** 2 wt %/BAlq(10 nm)/BPyTP(40 nm)/LiF (1 nm)/Al(100 nm)	–	–	3 743	21.5	[80]
**35**	ITO/HATCN/NPD/TrisPCz/CBP:**35** 20 wt % (10 nm)/CBP:**35** 6 wt % (20 nm)/BAlq/BPyTP/LiF/Al	–	–	5 600	15.3^c^	[82]
**37**	ITO/HATCN (10 nm)/NPD (40 nm)/TrisPCz (10 nm)/mCBP:**37** 10 wt % (25 nm)/mCBT (8 nm)/BPyTP (40 nm)/LiF (1 nm)/Al (100 nm)	–	–	4 929	17.8	[84]
**38**	ITO/HATCN (10 nm)/NPD (40 nm)/TAPC(10 nm)/26mCPy:**38** 6 wt % (25 nm)/DPPS (10 nm)/BmPyPB (40 nm)/LiF/Al	–	–	–	24.4	[85]
**39**	ITO/NPB (50 nm)/mCP (10 nm)/BCPO:**39** 10 wt % (20 nm)/DPEPO (10 nm)/TPBi (30 nm)/LiF (1 nm)/Al (100 nm)	11.0	10.8	10 676	9.7	[86]
**41**	ITO/HATCN (10 nm)/TAPC (40 nm)/TCTA (10 nm)/26mCPy:**41** 15 wt % (20 nm)/TmPyPB (45 nm)/Liq (2 nm)/Al (120 nm)	78.5	66.4	–	22.3	[88]
**44**	ITO/HATCN (10 nm)/TAPC (45 nm)/TCTA (10 nm)/CBP:**44** 30 wt % (20 nm)/TmPyPB (50 nm)/Liq (2 nm)/Al (110 nm)	58.0	51.6	–	16.5	[89]
**47**	ITO/HATCN (10 nm)/TAPC (40 nm)/TCTA/mCP:**47** 10 wt %/TmPyPb (40 nm)/Liq (2 nm)/Al (120 nm)	53.0	35.9	–	16.3	[90]
**50**	ITO/HATCN (10 nm)/TAPC (40 nm)/TCTA (10 nm)/26mCPy:**50** 15 wt % (20 nm)/TmPyPB (45 nm)/Liq (2 nm)/Al (120 nm)	83.0	63.8	–	22.9	[96]
**54b**	ITO/TAPC (40 nm)/mCP:**54b** 8 wt % (17 nm)/DPEPO (3 nm)/TmPyPB (50 nm)/LiF (0.8 nm)/Al (150 nm)	36.3	38.0	4 121	15.3	[97]
**57**	ITO/PEDOT (40 nm)/PVK:OXD-7:**57** 2 wt % (50 nm)/TPBI (30 nm)/Ba (4 nm)/Al (100 nm)	–	–	–	0.88	[102]
**59**	ITO/PEDOT (40 nm)/PVK:**59** 1 wt % (50–60 nm)/TPBi (30 nm)/Ba (4 nm)/Al	–	–	–	0.85	[110]

^a^Device peak values unless differently stated; ^b^recorded at 100 cd m^−2^; ^c^recorded at 1,000 cd m^−2^; ^d^device comprising light outcoupling structures; ^e^recorded at a current density of 10 mA cm^−2^.

## Conclusion

In conclusion, we have here reviewed the most recent trends in the field of phosphorescent platinum complexes, and their use as phosphors in light-emitting optoelectronic devices such as OLEDs. Indeed, such class of luminescent complexes still represents a fascinating research topic of enormous current interest, in particular in the case of derivatives with oxidation state +II. This is because these systems possess excellent photophysical properties that can be tuned by judicious molecular design through ligand modification. Seeking for emitters with improved features, interesting examples with great structural variety have been reported to date that are based not only on bidentate and tridentate moieties, but recently also on tetradentate scaffolds. Differently from many other transition metals, square planar platinum(II) complexes bearing π-conjugated ligands also possess a peculiar tendency to establish weak intermolecular interactions, such as metallophilic and π–π interactions. These additional features could further widen the already available chemical toolbox for designing highly efficient electrophosphorescent solid-state materials in the near future. Overall, design efforts have allowed the achievement of impressive OLED performances for devices embedding platinum-based triplet emitters with EQE above 30%. Such results have been achieved thanks to the combination of molecular and dipole moments orientation engineering in the electroactive thin film. Finally, recent reports on platinum(IV) derivatives demonstrate that this type of complexes do also possess interesting photophysics and therefore, further growing interest in their use as emitters in OLEDs could be also foreseen.
